# Multisocietal European consensus on the terminology, diagnosis, and management of patients with synchronous colorectal cancer and liver metastases: an E-AHPBA consensus in partnership with ESSO, ESCP, ESGAR, and CIRSE

**DOI:** 10.1093/bjs/znad124

**Published:** 2023-07-14

**Authors:** Ajith K Siriwardena, Alejandro Serrablo, Åsmund Avdem Fretland, Stephen J Wigmore, Jose Manuel Ramia-Angel, Hassan Z Malik, Stefan Stättner, Kjetil Søreide, Oded Zmora, Martijn Meijerink, Nikolaos Kartalis, Mickaёl Lesurtel, Kees Verhoef, Anita Balakrishnan, Thomas Gruenberger, Eduard Jonas, John Devar, Saurabh Jamdar, Robert Jones, Mohammad Abu Hilal, Bodil Andersson, Karim Boudjema, Saifee Mullamitha, Laurents Stassen, Bobby V M Dasari, Adam E Frampton, Luca Aldrighetti, Gianluca Pellino, Pamela Buchwald, Bengi Gürses, Nir Wasserberg, Birgit Gruenberger, Harry V M Spiers, William Jarnagin, Jean-Nicholas Vauthey, Norihiro Kokudo, Sabine Tejpar, Andres Valdivieso, René Adam

**Affiliations:** Hepato-Pancreato-Biliary Unit, Manchester Royal Infirmary, Manchester, UK; Department of Surgery, Miguel Servet University Hospital, Zaragoza, Spain; Department of Surgery, Oslo University Hospital, Oslo, Norway; Hepatobiliary and Liver Transplant Unit, Royal Infirmary of Edinburgh, Edinburgh, UK; Department of Surgery, University Hospital of Guadalajara, Guadalajara, Spain; Liver Surgery Unit, Royal Liverpool University Hospital, Liverpool, UK; Department of General, Visceral and Vascular Surgery, Salzkammergutklinikum, Vöcklabruck, Austria; Department of Surgery, Bergen University Hospital, Bergen, Norway; Department of Colorectal Surgery, Shamir Medical Centre, Tel Aviv, Israel; Department of Radiology, Amsterdam University Medical Centre, Amsterdam, the Netherlands; Department of Radiology, Karolinska Institutet, Stockholm, Sweden; Department of Surgery, Beaujon Hospital, Clichy, France; Department of Surgery, Erasmus University Medical Centre, Rotterdam, the Netherlands; Cambridge Hepato-Pancreato-Biliary Unit, Addenbrooke’s Hospital, Cambridge, UK; Department of Surgery, Hepatopancreatobiliary Centre, Health Network Vienna, Clinic Favoriten and Sigmund Freud University, Vienna, Austria; Department of Surgery, Groote Schuur Hospital, Cape Town, South Africa; Department of Surgery, Chris Hani Baragwanath Hospital, Johannesburg, South Africa; Hepato-Pancreato-Biliary Unit, Manchester Royal Infirmary, Manchester, UK; Liver Surgery Unit, Royal Liverpool University Hospital, Liverpool, UK; Department of Surgery, Poliambulanza Hospital, Brescia, Italy; Department of Surgery, Skane University Hospital, Lund, Sweden; Department of Hepatobiliary, Pancreatic and Digestive surgery, Hôpital Pontchaillou, Rennes, France; Oncology Department, Christie Hospital, Manchester, UK; Department of Surgery, Maastricht University Medical Centre, Maastricht, the Netherlands; Hepatobiliary and Liver Transplant Unit, Queen Elizabeth University Hospital, Birmingham, UK; Hepato-Pancreato-Biliary Unit, Royal Surrey County Hospital, Guildford, UK; Department of Surgery, Vita-Salute San Raffaele University and IRCCS San Raffaele Hospital, Milan, Italy; Department of Colorectal Surgery, Vall d’Hebron University Hospital, Universitat Autonoma de Barcelona UAB, Barcelona, Spain; Department of Advanced Medical and Surgical Sciences, Università degli Studi della Campania ‘Luigi Vanvitelli’, Naples, Italy; Department of Surgery, Skane University Hospital, Lund, Sweden; Department of Radiology, Koc University Medical Faculty, Istanbul, Turkey; Department of Surgery, Beilinson Hospital, Rabin Medical Centre, Tel Aviv University, Tel Aviv, Israel; Department of Medical Oncology and Haematology, Landesklinikum Wiener Neustadt, Wiener Neustadt, Austria; Cambridge Hepato-Pancreato-Biliary Unit, Addenbrooke’s Hospital, Cambridge, UK; Department of Surgery, Memorial Sloan Kettering Cancer Centre, New York, New York, USA; Department of Surgery, MD Anderson Cancer Centre, Houston, Texas, USA; Department of Surgery, National Centre for Global Health and Medicine, Tokyo, Japan; Department of Oncology, UZ Leuven, Leuven, Belgium; Hepatopancreatobiliary Surgery and Liver Transplant, HU Cruces, Bilbao, Spain; Hepatobiliary and Transplant Unit, Hôpital Paul Brousse, Paris, France

## Abstract

**Background:**

Contemporary management of patients with synchronous colorectal cancer and liver metastases is complex. The aim of this project was to provide a practical framework for care of patients with synchronous colorectal cancer and liver metastases, with a focus on terminology, diagnosis, and management.

**Methods:**

This project was a multiorganizational, multidisciplinary consensus. The consensus group produced statements which focused on terminology, diagnosis, and management. Statements were refined during an online Delphi process, and those with 70 per cent agreement or above were reviewed at a final meeting. Iterations of the report were shared by electronic mail to arrive at a final agreed document comprising 12 key statements.

**Results:**

Synchronous liver metastases are those detected at the time of presentation of the primary tumour. The term ‘early metachronous metastases' applies to those absent at presentation but detected within 12 months of diagnosis of the primary tumour, the term ‘late metachronous metastases’ applies to those detected after 12 months. ‘Disappearing metastases’ applies to lesions that are no longer detectable on MRI after systemic chemotherapy. Guidance was provided on the recommended composition of tumour boards, and clinical assessment in emergency and elective settings. The consensus focused on treatment pathways, including systemic chemotherapy, synchronous surgery, and the staged approach with either colorectal or liver-directed surgery as first step. Management of pulmonary metastases and the role of minimally invasive surgery was discussed.

**Conclusion:**

The recommendations of this contemporary consensus provide information of practical value to clinicians managing patients with synchronous colorectal cancer and liver metastases.

## Teaser

This consensus provides a practical framework for the clinician treating patients with synchronous colorectal cancer and liver metastases.

## Introduction

In 2020, the European Commission^1^ estimated that colorectal cancer accounted for 12.7 per cent of all new cancer diagnoses and 12.4 per cent of all deaths from cancer, making this the second most frequently occurring cancer. About one-fifth of patients with colorectal cancer have metastases either exclusively or predominantly in the liver at the time of presentation^2^. Hepatic metastases may also be detected later in the course of the disease^2^.

Current guidelines for the management of colorectal cancer are provided by the National Comprehensive Cancer Network (NCCN)^3^ and the European Society for Medical Oncology (ESMO)^4^ in addition to other organizations/societies^5–7^. The relative absence of high-quality evidence in relation to surgical aspects of the management of patients with synchronous colorectal cancer and liver metastases results in these guidelines providing only broad recommendations in this area. For example, neither the NCCN nor ESMO guidelines address definitions of synchronous/metachronous disease or disappearing metastases, and the focus on management is relatively limited in terms of guidance on selection of surgical treatment pathways^3,4^.

Recognizing this information gap, the Expert Group on OncoSurgery management of Liver Metastases (EGOSLIM)^8^ produced a report in 2015 on the management of patients with colorectal cancer and synchronous liver metastases. Almost a decade later, diagnostic options have increased and treatment pathways have become more complex^9^.

Consensus methodology is a valuable option to find concordance in current practice, considering both the difficulty in conducting high-quality surgical randomized trials in patients with synchronous colorectal cancer and liver metastases, and the persisting evidence of variation in the use of definitions for synchronous disease^10–12^. This project was a major, multiorganizational, multidisciplinary collaborative consensus to provide a practical framework for care of patients with synchronous colorectal cancer and liver metastases, with a focus on terminology, diagnosis, and management.

## Methods

### Overview and scope

This project was a multiorganizational, multidisciplinary consensus to produce a practical document to guide clinicians involved in the care of patients with synchronous colorectal cancer and liver metastases. The scope of the project was to review and, where necessary, update terminology and to describe current management pathways. The MEDLINE®, EMBASE, Web of Science and Cochrane databases were queried in July 2022. The search terms ‘colon cancer’, ‘rectum cancer’ and ‘liver metastases’ were used in combinations. A search was carried out to inform the construction of each statement before circulation. Separate searches were undertaken for terminology, diagnosis, composition of multidisciplinary team, considerations for ‘upfront’ synchronous surgery, chemotherapy for synchronous metastases, the ‘bowel-first’ and ‘liver-first’ approaches.

The final recommendations are based on expert consensus. Thus, the project report should not be considered as a comprehensive evidence review. Furthermore, although the consensus addresses integration of chemotherapy and (where appropriate) radiotherapy with surgery, the reader is referred to guidelines such as those of NCCN^3^ or ESMO^4^ for details on specific systemic chemo(radio)therapy regimens.

### Participants

This consensus project was commissioned by the executive committee of the European–African Hepato-Pancreato-Biliary Association (E-AHPBA) in September 2021. Formal submissions to participate in this project were accepted by the executive boards of the European Society of Surgical Oncology (ESSO), European Society of Coloproctology (ESCP), European Society of Gastrointestinal and Abdominal Radiology (ESGAR), and the Cardiovascular and Interventional Radiology Society of Europe (CIRSE). ESMO did not formally participate, but oncologists affiliated with this organization participated in the exercise. Consensus participants were selected through two routes: first, those who had published work in areas relevant to the consensus and, second, those who were invited to participate by their respective specialist societies. Participants in the consensus comprised 123 clinicians: hepatopancreatobiliary surgeons (including individuals with expertise in liver transplantation), colorectal surgeons, oncologists, radiologists (including individuals with expertise in MRI radiology and interventional radiology), cancer nurse specialists, histopathologists, and surgeons in training. Before the consensus, a series of qualitative interviews was undertaken with patients who had synchronous colorectal cancer and liver metastases, and their relatives/carers, to ascertain their views on management^13^. These patients’ views were used to inform the design of the questions and the subsequent statements of this consensus.

### Design and content of statements for consensus

A subgroup of the consensus participants representing surgical, oncological, and radiological specialties met in a series of online meetings to produce statements covering the scope of the project. Eighteen statements addressed definitions and clinical pathways. Specifically, these statements addressed terminology for synchronous and metachronous liver metastases, recommendations for the composition of a specialist multidisciplinary team (MDT), and tests required for diagnosis and management in both the emergency and elective settings. Management of patients with a synchronous presentation included statements on selection for systemic chemotherapy as first intervention, synchronous surgery, and the staged approach. Finally, there were specific statements on the term ‘disappearing metastases’, the role of minimally invasive surgery, and the management of pulmonary metastases. There were a series of qualifying sentences for each of the 18 statements, and a document with all statements was sent to all members of the consensus (*Appendix S1*).

### Consensus process

The consensus process took place between June 2022 and December 2022, and consisted of two rounds of a Delphi process followed by a final face-to-face meeting^14,15^. The Delphi process used SurveyMonkey (https://www.surveymonkey.com/mp/audience). The first round took place in September 2022. Results were collated and a threshold of 70 per cent was set for consensus. Statements for which there was less than 70 per cent support were removed or modified with respondents’ feedback, and used to produce a second round of the Delphi process. The third component was a final face-to-face meeting held in Zaragoza, Spain, on 2 and 3 December 2022. The second-round statements, together with the results of the second round of Delphi voting, were then discussed individually, followed by an audience vote.

### Assembly of consensus report

All involved in the consensus process were invited to participate in the writing process. Results from the two Delphi rounds together with information from the face-to-face meeting were integrated into a final series of 12 statements. Iterations were shared by electronic mail to arrive at a final agreed document comprising 12 statements. Areas of persisting disagreement (lack of consensus) were also noted and acknowledged in the final document. As the recommendations were by consensus, grading of evidence^16^ was not used. The 18 statements used at the outset were compressed to 12 key statements after discussion. Research proposals generated during the consensus process were collated and will take the form of a separate manuscript.

Before submission, the final document was reviewed by a validation committee comprising experts on this topic (W. Jarnagin, J.-N. Vauthey, N. Kokudo, and S. Tejpar).

### Role of sponsors in consensus process

Multiple sponsors contributed to support the face-to-face consensus meeting. None had any role in the design of the statements or in the recommendations made in the final report.

### Ethics

The E-AHPBA Scientific and Research Committee reviewed and approved this study. Although the project involves a collaboration between E-AHPBA, ESSO, ESCP, ESGAR, and CIRSE, the responsibility for the views expressed in this manuscript rests with the consensus authors and this document does not represent an official position statement of any organization.

### Planned review and renewal

It is the intention to update this consensus document approximately 5 years after publication.

## Results

### Terminology for description of synchronous and metachronous liver metastases

Synchronous liver metastases are defined as those detected at the time of presentation of the primary tumour (colonic or rectal cancer) (*Table 1*). Use of this term is unchanged from the EGOSLIM consensus^8^. Evidence of differential survival between patients with liver metastases detected in the first 12 months after diagnosis of the primary tumour compared with those detected after the first year is recognized by retention of the terms ‘early’ and ‘late’ metachronous metastases respectively^8^. To be termed ‘metachronous disease’, liver metastases should be excluded on cross-sectional imaging at the time of diagnosis of the primary tumour.

**Table 1:** E-AHPBA/ESSO/ESCP/ESGAR/CIRSE consensus terminology for synchronous and metachronous liver metastases

Liver metastases detected at the time of diagnosis of the primary are termed ‘synchronous’
The definition of synchronous liver metastases also includes patients with incidental liver metastases detected during surgery
To be termed ‘metachronous disease’, liver metastases should have been excluded on cross-sectional imaging at the time of diagnosis of the primary tumour
Liver metastases detected up to 12 months after diagnosis of the primary tumour—but absent at presentation—are termed ‘early metachronous metastases’
Liver metastases detected more than 12 months after diagnosis of the primary are termed ‘late metachronous metastases’

E-AHPBA, European-African Hepato-Pancreato-Biliary Association;ESSO, European Society of Surgical Oncology; ESCP, European Society of Coloproctology; ESGAR, European Society of Gastrointestinal and Abdominal Radiology; CIRSE, Cardiovascular and Interventional Radiology Society of Europe.

Consideration was given to extending the time interval for use of the term ‘synchronous’ to either 3 or 6 months after diagnosis of the primary tumour^17^. Evidence from a literature review indicating similar survival for patients with synchronous liver metastases was also considered as the recommendations contradict the EGOSLIM recommendations^17^. However, from a practical perspective, management of the primary tumour will likely have taken place before the 3- or 6-month extended cut-offs, and thus treatment of liver metastases discovered at these later time points is in effect the management of early metachronous disease. Therefore, this consensus does not recommend these extended time intervals for use of the term ‘synchronous’.

### Scope and constitution of a multidisciplinary team (tumour board) for management of patients with synchronous colorectal cancer and liver metastases

The consensus recommends that all patients with liver metastases from colorectal cancer should have their care reviewed at a specialist MDT with expertise in the management of liver metastases^18,19^.

The consensus recommends that such an MDT should include the following core specialties: radiology (with expertise in gastrointestinal imaging), interventional radiology, hepatobiliary (liver) surgery, colorectal surgery, gastrointestinal oncology, radiation oncology, histopathology, cancer nurse specialist, and MDT coordinator (case manager). In addition to this core group, extended membership could comprise (but not be restricted to) interventional radiology, radiation oncology/radiotherapy, thoracic surgery, liver anaesthetics, and gastroenterology. The consensus acknowledges that the role and availability of cancer nurse specialists varies between healthcare systems. The consensus also accepts that, in practice, the composition of an MDT represents a compromise between an ideal arrangement, including both core and extended members, and a pragmatic acknowledgement that logistics and workforce issues often restrict the ability of all specialties to be present in a single meeting.

### Diagnostic tests

The consensus recommendations broadly follow those of NCCN^3^ and ESMO^4^, and state the following:

- Contrast-enhanced CT of the thorax, abdomen and pelvis should be undertaken at the time of presentation.
- Liver MRI with hepatobiliary contrast agents should be undertaken at the time of presentation (and before any chemotherapy)^20^. If hepatobiliary contrast agents are not available, standard liver agents (not hepatocyte-specific) may be used.
- There should be histological confirmation of diagnosis from biopsy of the primary tumour but not ordinarily from liver metastases.
- Consideration should be given to undertaking a complete endoscopic examination of the colon and rectum at the time of diagnosis. CT colonography can be undertaken if complete endoscopy cannot be performed^21^.
- Where available, MRI for low and mid rectal primary tumours (within 12 cm proximal to the anal verge) should be undertaken at the time of presentation^20^. Transrectal ultrasound imaging may be an alternative, although MRI is preferred^22^.
- Determination of mutation status for *RAS*, *BRAF*, and *HER2* amplifications, either individually or as part of a next-generation sequencing panel, together with determination of mismatch repair status, should be performed from the primary tumour^5,23,24^.
- Lesional liver biopsy may need to be considered in some specific settings, for example, if there is a history of a different malignancy.
- The tumour marker carcinoembryonic antigen (CEA) should be measured at baseline presentation for disease monitoring/surveillance^25^.

The consensus acknowledges the value of [^18^F]fluorodeoxyglucose PET in decision-making in patients with stage IV colorectal cancer, but does not recommend such imaging to be used routinely in the diagnosis of patients with synchronous colorectal cancer and liver metastases^3,26^.

The consensus also acknowledges that mutation analysis is currently not available in many healthcare systems.

### Clinical management of the patient with synchronous colorectal cancer and liver metastases and an emergency presentation

The consensus recommends that surgery aimed at addressing emergency complications of the primary tumour should be considered after appropriate resuscitation in patients with a performance status that permits active treatment^27^. There should be no intervention directed at the liver metastases during the emergency presentation.

The consensus recommends consideration of an endoluminal stent, defunctioning stoma or resectional surgery for patients with intestinal obstruction, depending on the circumstances and available expertise^27,28^.

In selected patients presenting with bleeding from rectal tumours, radiotherapy or interventional radiology techniques can be considered^29,30^.

Complete diagnostic staging should be undertaken after recovery from the acute episode.

### Further clinical assessment of the patient with synchronous colorectal cancer and liver metastases and an elective presentation

In addition to the diagnostic tests above, assessment of fitness for intervention is recommended. Additional fitness tests are not routinely recommended for patients with Eastern Cooperative Oncology Group 0 status^31^. The consensus recommends that, where available, dynamic cardiopulmonary exercise testing could be considered before surgery, with selection depending on performance status^32^. A prehabilitation programme could also be considered, depending on availability and time to surgical intervention^33^. The consensus acknowledges the limited evidence for prehabilitation programmes at the present time.

Accurate documentation of disease stage and distribution is recommended as part of the detailed clinical assessment after completion of diagnostic tests and the consensus recommends documentation (which could be on a standardized pro forma) as follows:

- In relation to the primary tumour, sidedness and radiological assessment of T category (including circumferential margin involvement) and nodal status should be recorded.
- The presence or absence of extrahepatic metastases should be specified together with site.
- In relation to thoracic metastases, number, laterality, and definite or ‘indeterminate’ should be noted.
- In relation to liver metastases, the size, number, and distribution within Couinaud segments should be specified. The consensus acknowledges that, although documentation of extent, size, and distribution of liver metastases is an important component of assessment, this can be challenging in the situation of patients with multiple liver metastases. In this situation, the consensus acknowledges that relevant practice would be to document the segments involved at baseline as this could have practical implications for any potential surgical treatment after induction systemic therapy^34,35^. The consensus does not define a threshold number of metastases above which the benefit of documenting the number and size of lesions is limited.

During the Delphi rounds, statements were also included on describing the location of liver metastases in relation to important inflow/outflow structures and the vena cava. Although these were not retained in the final recommendations, the consensus notes that there may be situations in which description of critical structures adjacent to a tumour would be valuable.

### Considerations for ‘upfront’ synchronous resection of liver tumour(s) and bowel primary tumour in patients with resectable synchronous colorectal cancer and liver metastases

In this consensus, the term ‘upfront’ applies to a proposed intervention when it is the first treatment. Synchronous resection of synchronous disease is defined as resection of liver metastases and the primary bowel tumour under a single general anaesthetic (single surgery).

This consensus acknowledges the practical distinction between the management of colonic and rectal primary tumours, including in relation to the use of neoadjuvant radiotherapy^36,37^. The consensus acknowledges the evidence of differential biological behaviour according to the sidedness or laterality of colonic cancer, but notes that, at the present time, this information is not integrated widely into treatment planning^38^.

The consensus makes the following recommendations in relation to undertaking synchronous hepatectomy with colectomy in patients with colonic tumours:

- Although synchronous resection of liver and colonic tumours as a first step is supported by the consensus, it is emphasized that, for most patients with colorectal cancer and synchronous liver metastases, systemic chemotherapy and not surgery will be the preferred first treatment^3,4^.
- The consensus recommends that, for patients considered for synchronous hepatectomy and colectomy, there should be a combination of adequate functional volume in the future liver remnant and a primary colonic tumour not requiring neoadjuvant systemic treatment. The consensus does not define adequate future liver remnant beyond emphasizing that there must be adequate biliary drainage, portal and arterial inflow, adequate venous drainage, and sufficient parenchymal volume in the future remnant liver^39,40^.
- The consensus recommends that, when upfront synchronous liver resection is to be undertaken together with colectomy, the liver resection component should be a minor hepatectomy^41^.

No consensus was reached on whether to support upfront synchronous major hepatectomy with colectomy, although it is acknowledged that this combination can be undertaken safely^11,42^.

For patients with a rectal primary tumour, the consensus does not recommend upfront, synchronous liver resection together with rectal surgery. These patients normally require non-surgical treatments as a first step, including radiotherapy or chemoradiotherapy/total neoadjuvant therapy^43,44^.

The consensus acknowledges that the results of the ongoing COLLISION^45^ and NEW-COMET^46^ trials comparing ablation with resection may influence a change towards the use of the term ‘locally treatable’ rather than exclusively ‘resectable’,. Options for local treatment should be considered during case discussion by a MDT.

### Considerations for ‘upfront’ systemic chemotherapy in patients with synchronous colorectal cancer and liver metastases

The consensus recommends systemic chemotherapy as a first treatment in patients with a performance status that precludes surgery (but not systemic chemotherapy), in those with extrahepatic disease at presentation (M1b status), and in patients with peritoneal metastases at presentation (M1c status)^3,4^. The consensus refers clinicians to the current NCCN^3^ and ESMO^4^ guidelines for decision-making around choice of chemotherapy agents, use of combination chemotherapy, biological agent(s), and treatment regimens.

### Considerations for a ‘bowel-first’ approach in patients with synchronous colorectal cancer and liver metastases

The consensus supports the ‘bowel-first’ approach in two settings: first, the patient with a symptomatic primary tumour and/or imminent intestinal obstruction or perforation^21^, and second, as part of a staged approach (bowel first, liver second) to tumour clearance in patients with synchronous disease treated by systemic chemotherapy^47^. The consensus recommends restaging with repeat cross-sectional imaging of the thorax, abdomen, and pelvis, and further MDT discussion between surgical stages. Resection of an asymptomatic primary colorectal tumour is not recommended in the presence of non-resectable liver metastases^48,49^.

### Considerations for a ‘liver-first’ approach after systemic chemotherapy in patients with synchronous colorectal cancer and liver metastases

The term ‘liver first’ is defined in this consensus as liver resection as the first surgical intervention in patients with synchronous colorectal cancer and liver metastases^50^.

The consensus supports the liver-first approach in the following situations:

- When there are specific liver-related criteria, such as borderline resectability, which favour hepatectomy first after systemic chemotherapy^51,52^.
- Patients with rectal tumours with a response to chemoradiotherapy. Liver resection can be undertaken in the window between completion of chemo(radio)therapy of the rectal cancer and the ensuing evaluation of treatment response before surgical treatment of the rectal primary tumour^52,53^. This is the most widely accepted indication for the liver-first approach. Attention should be given to avoiding the liver-first approach in patients with locally advanced, surgically unresectable primary tumours.
- Patients with rectal cancer and resectable synchronous liver metastases who have a clinical complete response of the primary tumour to neoadjuvant treatment^54,55^. In this setting, it is possible that the liver-first approach may evolve into a liver surgery-only approach for the patient.

### Terminology and management of ‘disappearing’ liver metastases

The consensus first addresses the terminology in this situation (*Table 2*). The consensus recommends hepatobiliary contrast-enhanced MRI before and after systemic chemotherapy to assess for ‘disappearing’ lesions as this is in keeping with current state-of-the-art liver imaging^56,57^. It is accepted that this 2022 consensus terminology is dependent on MRI and not all clinicians have access to this. There was no consensus to state that complete response on CT alone could justify the term ‘disappearing metastases’.

**Table 2:** E-AHPBA/ESSO/ESCP/ESGAR/CIRSE consensus definitions of disappearing liver metastases

The term ‘disappearing metastases’ is defined in this study as lesions present on baseline contrast MRI which are no longer visible on hepatobiliary contrast MRI after systemic chemotherapy
The presence of a scar on cross-sectional imaging is termed ‘evidence of treatment response’ but, if visible on hepatobiliary contrast MRI, the lesion is not regarded as disappearing

E-AHPBA, European-African Hepato-Pancreato-Biliary Association;ESSO, European Society of Surgical Oncology; ESCP, European Society of Coloproctology; ESGAR, European Society of Gastrointestinal and Abdominal Radiology; CIRSE, Cardiovascular and Interventional Radiology Society of Europe.

No consensus was reached on the use of an observation policy in patients with a complete radiological response to systemic chemotherapy in liver metastases as assessed on MRI. CEA measurement (that is a relevant biochemical response/decline in serum values) can be used to augment clinical decision-making in patients who have a response to treatment, provided that baseline, pretreatment values are available for comparison^25^.

### Management of synchronous pulmonary metastases in patients with synchronous colorectal cancer and liver metastases

The consensus regards the presence of definite pulmonary metastases on cross-sectional imaging (M1b disease) to be an indication for systemic treatment as first line rather than surgery^3,4^.

The consensus does not support resection of pulmonary metastases at the same time as resection of liver tumours and/or the colonic primary tumour.

The consensus recommends that the opinion of a thoracic MDT about the potential locoregional treatment of pulmonary metastases should be sought before embarking on liver or bowel surgery in patients with suspected or confirmed pulmonary metastases.

### Role of minimally invasive surgery in management of colorectal cancer with synchronous liver metastases

The consensus regards minimally invasive approaches for both primary tumour and liver metastases as appropriate options^58–60^. The consensus acknowledges that, to date, the published literature has focused predominantly on minimally invasive hepatectomy rather than on the management of patients with synchronous colorectal cancer and liver metastases using laparoscopic or robotic approaches.

## Discussion

This consensus represents arguably the most comprehensive exercise undertaken to date to address the management of patients with synchronous colorectal cancer and liver metastases. It is the first to bring together multiple professional societies to address this topic. Despite the scope and extent of this consensus, there are important factors that could have influenced the recommendations and introduced bias, and these should be discussed^61^. First, the composition of the consensus group was predominantly surgical, with hepatobiliary surgeons constituting the largest individual group. Although this was necessary to have sufficient expertise and depth to address complex surgical pathways, it could have introduced bias towards operative interventions. Second, the consensus does not address the likely impact of the molecular genetics of colorectal cancer on surgical decision-making, for example, the evidence of poor outcome after hepatectomy in patients who carry the *BRAF* V600E mutation^62^. However, mutation analysis may not be available at the outset of management and is not available at all for many patients in a global context. Third, some aspects of the recommendations of this consensus are biased against healthcare systems with limited access to MRI. For example, the decision to use MRI in the definition of disappearing liver metastases could restrict the utility of this definition.

Having reviewed these limitations, what can be gained from this consensus? The focus towards standardizing the definitions of synchronous and metachronous disease is an important cornerstone of this project.

The terminology around the use of the terms ‘synchronous’, ‘early metachronous’ and ‘late metachronous’ is retained because of evidence favouring these descriptors. This terminology should be adopted universally for disease description and comparison of outcomes.

The definition of ‘disappearing’ metastases takes an important step towards integration of modern imaging by relying on MRI.

The consensus then follows the treatment pathway of a patient with colorectal cancer and synchronous liver metastases, starting with a focus on the composition of the MDT. Here, the recommendations on core and extended members do not differ substantially from those described in both NCCN and ESMO guidelines. Similarly, as would be expected, the consensus recommendation on diagnostic tests closely follows both NCCN and ESMO recommendations.

In terms of management, the consensus provides guidance on the use of systemic chemotherapy as first treatment, synchronous surgery (as a first intervention), and staged surgery.

The lack of consensus about the role of major hepatectomy combined with colonic surgery is highlighted. Although major hepatectomy can be combined with colonic resection, the evidence is predominantly based on case series or retrospective data and insufficient to make a recommendation^11,63,64^.

The consensus regarded minimally invasive approaches or open surgery to either the primary tumour or liver metastases as equivalent. It is of note that the literature on minimally invasive liver surgery does not focus on patients with synchronous disease. To this extent, the forthcoming Internationally Validated European Guidelines Meeting on Minimally Invasive Liver Surgery (IEGUMILS) 2024 will have a specific focus on this area, and on current research in patients with synchronous colorectal cancer and liver metastases (M. Abu Hilal, personal communication).

Setting the findings in the context of current evidence and guidelines, the consensus approach permitted the flexibility to focus on important practical aspects of management. For example, although the importance of accurate documentation of disease distribution is discussed, this is thought to be the first document to address the situation of the patient with multiple liver metastases in all segments, in whom the distribution of disease is more relevant to future management than a numerical or volume-based description of tumour burden.

In summary, this multisociety, multidisciplinary consensus provides information of practical value to clinicians treating patients with synchronous colorectal cancer and liver metastases. The clarifications of terminology can be adopted generally and would help in future comparison of outcomes. The clinical recommendations emphasize the importance of comprehensive staging, the need to integrate systemic treatments with surgery, and current areas of equipoise and limitations in knowledge. Incorporation of knowledge on the cancer biology of colorectal cancer into management, together with an understanding of the genetic heterogeneity of metastatic colorectal cancer, will likely help to rationalize future management^65,66^.

## Collaborators

Joint E-AHPBA/ESSO/ESCP/ESGAR/CIRSE 2022 Consensus on colorectal cancer with synchronous liver metastases: Hauke Lang^37^, Martin Smith^17^, Michelle L deOliveira^38^, Anya Adair^4^, Stefan Gilg^39^, Rutger-Jan Swijnenburg^40^, Joris Jaekers^41^, Santhalingam Jegatheeswaran^1^, Carlijn Buis^42^, Rowan Parks^4^, Maximilian Bockhorn^43^, Thierry Conroy^44^, Panagiotis Petras^45^, Florian Primavesi^7^, Anthony K. C. Chan^1^, Federica Cipriani^25^, Laura Rubbia-Brandt^46^, Lucy Foster^47^, Amr Abdelaal^48^, Sheraz Yaqub^3^, Nuh Rahbari^49^, Constantino Fondevila^50^, Manuel Abradelo^51^, Niels FM Kok^52^, Luis Tejedor^53^, Dario Martinez-Baena^54^, Daniel Azoulay^36^, Manuel Maglione^55^, Mario Serradilla-Martín^2^, José Azevedo^56^, Fabrizio Romano^57^, Pål-Dag Line^3^, Teresa Abadía Forcén^2^, Yves Panis^58^, Nicolas Stylianides^59^, Reto Bale^60^, Emilio Quaia^61^, Nuha Yassin^62^, Victoria Duque^2^, Eloy Espin-Basany^26^, Jarno Mellenhorst^22^, Adam Rees^59^, Ademola Adeyeye^63^, Jurriaan B. Tuynman^64^, Constantinos Simillis^65^, Sarah Duff^59^, Richard Wilson^66^, Paola De Nardi^67^, Gabriella Jansson Palmer^68^, Andee Dzulkarnaen Zakaria^69^, Teresa Perra^70^, Alberto Porcu^70^, Nicolò Tamini^71^, Michael E. Kelly^72^, Islam Metwally^73^, Stefan Morarasu^74^, Fabio Carbone^75^, Mercedes Estaire-Gómez^76^, Elena Martin Perez^77^, Jennifer Seligmann^78^, Simon Gollins^79^, Michael Braun^21^, Amelia Hessheimer^80^, Vincente Alonso^81^, Ganesh Radhakrishna^21^, Noreen Alam^21^, Constantinos Camposorias^21^, Jorge Barriuoso^21^, Paul Ross^82^, Ahmed Ba-Ssalamah^83^, Sivakumar Muthu^84^, Rafik Filobbos^84^, Vinotha Nadarajah^84^, Annas Hattab^84^, Claire Newton^1^, Sharon Barker^1^, Jill Sibbald^1^, Jodie Hancock^1^, Nicola de Liguori Carino^1^, Rahul Deshpande^1^, Francesco Lancellotti^1^, Sandra Paterna^2^, Marta Gutierrez-Diez^2^, Consuelo Artigas^2^.

## Collaborator institutional affiliations

^37^General, Visceral and Transplant Surgery, University Hospital Mainz, Mainz, Germany; ^38^University Hospital Zurich, Division of HPB and Transplant Surgery Zurich, Switzerland; ^39^Department of Surgery, CLINTEC, Department of surgery and oncology, Karolinska Institutet, Stockholm, Sweden; ^40^Department of Surgery, Amsterdam UMC, University of Amsterdam, Cancer Center Amsterdam, The Netherlands; ^41^Department of Visceral Surgery, UZ Leuven, Leuven, Belgium; ^42^Hepatobiliary and Liver transplantation unit, University of Groningen, Groningen, Netherlands; ^43^General and Visceral Surgery University Hospital Oldenburg, Oldenburg, Germany; ^44^Medical Oncology Department, Institut de Cancérologie de Lorraine, Vandœuvre-lès-Nancy, France; ^45^Ippokratio University Hospital of Thessaloniki, 5th Surgical Department of Aristotle University, Thessalonki, Greece; ^46^Department of Pathology and Immunology, University of Geneva, Geneva, Switzerland; ^47^Department of Histopathology, Manchester University NHS Foundation Trust, Manchester, UK; ^48^HPB surgery and Liver transplantation, Ain Shams University, Cairo, Egypt; ^49^Department of Surgery, University Hospital Mannheim, Mannheim, Germany; ^50^Department of Hepatobiliary Surgery and Transplantation, Hospital Universitario La Paz, Madrid, Spain; ^51^Department of Surgery, 12 de Octubre University Hospital, Madrid, Spain; ^52^Department of Surgical Oncology, Netherlands Cancer Institute, Amsterdam, The Netherlands; ^53^Department of Surgery, Hospital Punta de Europa, Algeciras, Spain; ^54^Hospital Universitario Nuestra Señora de Valme, HPB Surgery Unit, Valme University Hospital, Seville, Spain; ^55^Department of Visceral, Transplant, and Thoracic Surgery, Medical University of Innsbruck, Innsbruck, Austria; ^56^Colorectal Department, Digestive Unit, Champalimaud Center for the Unknown, Champalimaud Foundation, Portugal; ^57^Università Milano Bicocca, HPB Surgery, Fondazione IRCCS San Gerardo Monza, Italy; ^58^Colorectal Surgery Center Institut des MICI, Digestive surgery à Neuilly, Groupe Hospitalier Privé Ambroise, Paris, France; ^59^Department of Colorectal Surgery, Manchester University NHS Foundation Trust, Manchester, UK; ^60^Department of Radiology and Interventional Oncology, Medical University, Innsbruck, Austria; ^61^Department of Radiology, Medical University of Padova, Padova, Italy; ^62^Department of Colorectal Surgery, Royal Wolverhampton Hospital, Wolverhampton, UK; ^63^Department of Surgery, Afe Babalola University, Nigeria; ^64^Department of Colorectal Surgery, Amsterdam Medical Center, Amsterdam, Netherlands; ^65^Department of Colorectal Surgery, Addenbrooke's Hospital, Cambridge, UK; ^66^Department of Oncology, Beatson West of Scotland Cancer Centre, Glasgow, UK; ^67^Department of Gastrointestinal Surgery, San Raffaele Scientific Institute, Milan, Italy; ^68^Division of Coloproctology, Karolinska University hospital; MMK, Karolinska Institutet, Sweden; ^69^Department of Surgery, School of Medical Sciences & Hospital USM, Universiti Sains Malaysia, Kelantan, Malaysia; ^70^Department of Medicine, Surgery and Pharmacy, University of Sassari, Sassari, Italy; ^71^Colorectal Surgery Unit, IRCCS San Gerardo dei Tintori, Monza, Italy; ^72^St James Hospital Dublin, School of Medicine, Trinity College Dublin, Dublin, Ireland; ^73^Oncology Center Mansoura University (OCMU), Mansoura City,Egypt; ^74^2nd Department of Surgical Oncology, Regional Institute of Oncology, and Grigore T Popa University of Medicine and Pharmacy, Iasi, Romania; ^75^Department of Digestive Surgery, European Institute of Oncology IRCCS, Milan, Italy; ^76^Department of Colorectal Surgery, Severo Ochoa Hospital, Leganés, Spain; ^77^Department of General and Digestive Surgery, University Hospital de La Princesa, Madrid, Spain; ^78^Department of Gastrointestinal Oncology, St. James's University Hospital, Leeds, UK; ^79^North Wales Cancer Treatment Center, Bangor, Wales; ^80^Department of General Surgery, University Hospital La Paz, Madrid, Spain; ^81^Department of Medical Oncology, Miguel Servet, Hospital, Zaragoza, Spain; ^82^Department of Oncology, Guy's and St. Thomas’ Hospital, London, UK; ^83^Department of Biomedical Imaging and Image-guided Therapy, General Hospital of Vienna (AKH), Medical University of Vienna, Vienna, Austria; ^84^Department of Radiology, Manchester University NHS Foundation Trust.

## Supplementary Material

znad124_Supplementary_Data

## Data Availability

There are no additional data to make available in addition to the consensus statements.
