# Supplementary material for: Multisocietal European consensus on the terminology, diagnosis, and management of patients with synchronous colorectal cancer and liver metastases: an E-AHPBA consensus in partnership with ESSO, ESCP, ESGAR, and CIRSE
Source: Br J Surg. 2023 Jul 14;110(9):1161–70. doi: 10.1093/bjs/znad124 (PMC10416695; doi:10.1093/bjs/znad124)
Supplement: znad124_Supplementary_Data [file znad124_supplementary_data.docx]

**The multi-societal European consensus on the terminology, diagnosis and management of patients with synchronous colorectal cancer and liver metastases: an E-AHPBA^1^ consensus in partnership with ESSO^2^, ESCP^3^, ESGAR,^4^ and CIRSE^5^.**

**Abbreviations:**

**^1^E-AHPBA:** European-African Hepato-Pancreato-Biliary Association;

**^2^ESSO:**European Society of Surgical Oncology;

**^3^ESCP:** European Society of Coloproctology;

**^4^ESGAR:**European Society of Gastrointestinal and Abdominal Radiology;

**^5^CIRSE:** Cardiovascular and Interventional Radiology Society of Europe.

**Ajith K Siriwardena^1^, Alejandro Serrablo^2^, Åsmund Avdem Fretland^3^, Stephen J Wigmore^4^, Jose Manuel Ramia-Angel^5^ ,Hassan Z Malik^6^, Stefan Stättner^7^, Kjetil Søreide^8^ ,Oded Zmora ^9^, Martijn Meijerink^10^, Nikolaos Kartalis^11^, Mickaёl Lesurtel^12^, Kees Verhoef^13^, Anita Balakrishnan ^14^, Thomas Gruenberger ^15^, Eduard Jonas ^16^ , John Devar ^17^ , Saurabh Jamdar ^1^ ,Robert Jones^6^, Mohammad Abu Hilal ^18^ , Bodil Andersson^19^ ,Karim Boudjema ^20^, Saifee Mullamitha ^21^, Laurents Stassen^22^ , Bobby VM Dasari^23^, Adam E Frampton^24^, Luca Aldrighetti^25^, Gianluca Pellino^26^, Pamela Buchwald^19^, Bengi Gürses^27^, Nir Wasserberg^28^, Birgit Gruenberger^29^, Harry VM Spiers^14^, William Jarnagin^30^, Jean-Nicholas Vauthey^31^, Norihiro Kokudo^32,^ Sabine Tejpar ^33^, Andres Valdivieso ^34^ and René Adam^35^ on behalf of the joint E-AHPBA, ESSO, ESCP, ESGAR, CIRSE 2022 Consensus on colorectal cancer with synchronous liver metastases*.**

**Corresponding author:** Professor Ajith K Siriwardena MD FRCS

Hepato-Pancreato-Biliary Unit

Manchester Royal Infirmary

Oxford Road, Manchester M13 9WL.

Email: [ajith.siriwardena@mft.nhs.uk](mailto:ajith.siriwardena@mft.nhs.uk)

**Supplementary Materials - Index**

| **Supplementary Appendixes** |  |
| --- | --- |
| Appendix S1 | *page 3* |
|  |  |

**Supplementary Appendixes**

**APPENDIX S1: STATEMENTS FOR FIRST ROUND OF DELPHI VOTING**

**SECTION 1: TERMINOLOGY.**

Question 1: What is the optimal definition of the term “synchronous” liver metastases?

(Current concepts of the cancer biology of liver metastasis in colorectal cancer indicate that all metastases may be synchronous but present clinically at variable time points in the disease course. Thus, the definition of synchronous disease should be clinical and help to focus optimal management).

1. Liver metastases present at the time of diagnosis of the primary.
2. The definition should also include patients with incidental liver metastases detected intra-operatively.
3. Alternatively, the timeframe of “synchronous” should be increased from that of the EGOSLIM consensus 2015 to include liver metastases discovered up to 6 months after diagnosis of the primary tumour.

Question 2: What are the optimal definitions of “metachronous” liver metastases?

1. To be termed “metachronous” disease, liver metastases should ideally have been excluded on cross-sectional imaging at the time of diagnosis of the primary tumour.
2. Liver metastases detected up to 12 months after diagnosis of the primary tumour (but absent at presentation) should be termed “Early Metachronous” metastases.
3. Alternatively, if the 6-month timeframe for the term synchronous is to be adopted then “early metachronous” refers to lesions discovered after 6 months and before 12 months.
4. Liver metastases detected more than 12 months after diagnosis of the primary are termed “Late metachronous metastases.”

**SECTION 2: DIAGNOSIS.**

Question 3: What tests are required at the time of diagnosis of a patient with colorectal cancer and synchronous liver metastases?

1. There should be biopsy confirmation from the primary tumour.
2. A complete endoscopic examination of the colon and rectum should be performed at the time of diagnosis. A CT (virtual) colonoscopy could be undertaken if complete endoscopy cannot be performed.
3. Molecular profiling including RAS and MSI as a minimum should be performed from the primary tumour in all patients where feasible to aid in further management.
4. Biopsy of liver metastases to confirm diagnosis is not ordinarily required.
5. Lesional liver biopsy may need to be considered in some specific settings – for example if there is a prior history of a different malignancy.
6. Contrast-enhanced computed tomography (CT) of the thorax, abdomen and pelvis should be undertaken at time of presentation.
7. Where available, hepatobiliary contrast-enhanced magnetic resonance scan (MR) of the liver should be undertaken at the time of presentation (and prior to any chemotherapy).
8. Where available, MR for low and mid rectal primary tumours (below 12 cm from the anal verge) should be undertaken at the time of presentation. Trans rectal ultrasound (TRUS) may be an alternative. A structured reporting template for MR scans and TRUS of the rectum should be used.
9. ^18^Fluoro-deoxyglucose positron emission tomography (FDG-PET) should be undertaken at the time of presentation in patients with colorectal cancer and synchronous liver metastases.
10. The tumour marker carcino-embryonic antigen (CEA) should be measured at baseline presentation for disease monitoring/surveillance.
11. The tumour marker carbohydrate antigen CA 19-9 should be measured at baseline presentation for disease monitoring/surveillance.

**SECTION 3: INITIAL MANAGEMENT - THE URGENT PRESENTATION.**

Question 4: When a patient presents as an emergency (with perforation, obstruction or life-threatening bleeding) with colorectal cancer and synchronous liver metastases and a performance status which permits active treatment (with resuscitation) what should be the initial management?

1. Surgery aimed at addressing the emergency complication of the primary tumour should be considered.
2. Intra-operatively detected liver metastases should NOT be biopsied.
3. There should be no intervention directed at the liver metastases during the urgent presentation.
4. A diverting stoma (with no resection of the primary) or resectional surgery can both be considered for patients with intestinal obstruction, depending on the tumour location, available expertise and patient status.
5. Bowel stenting could be performed as a bridge to surgery in selected patients if expertise is available, but perforation can potentially worsen long-term outcomes.
6. Patients with rectal tumours who present with bleeding may be treated with radiotherapy.
7. Patients with colorectal tumours who present with bleeding may be treated with interventional radiological procedures.

**SECTION 4: ELECTIVE MANAGEMENT OF SYNCHRONOUS DISEASE.**

**LIVER METASTASES SPECIALIST MULTIDISCIPLINARY TEAM.**

Question 5: Should all patients with liver metastases from colorectal cancer have their care reviewed at an MDT with expertise in the surgical management of liver metastases?

1. Yes – all patients with liver metastases from colorectal cancer should have their care reviewed at a specialist MDT with expertise in the surgical management of liver metastases.
2. No – patients should only be referred if they have M1a disease and if their performance status permits active treatment.

**SECTION 4: ELECTIVE MANAGEMENT OF SYNCHRONOUS DISEASE.**

**LIVER METASTASES SPECIALIST MULTIDISCIPLINARY TEAM.**

Question 6: Which specialties should (ideally) be represented in a specialist liver metastases multidisciplinary team/tumour board?

1. Radiologist with an expertise in gastrointestinal imaging.
2. Interventional Radiologist.
3. Hepatobiliary Surgeon.
4. Colorectal Surgeon.
5. Thoracic Surgeon.
6. Liver transplant Surgeon.
7. Liver anaesthesiologist.
8. Gastrointestinal oncologist.
9. Radiation oncologist.
10. Gastrointestinal physician.
11. Cancer specialist nurse.
12. Dietitian.
13. Palliative care physician.
14. MDT co-ordinator (case manager).

**SECTION 5: INITIAL CLINICAL ASSESSMENT IN PATIENTS WITH POTENTIALLY RESECTABLE SYNCHRONOUS DISEASE WITH AN ELECTIVE PRESENTATION.**

Question 7: In addition to detailed history, physical examination, baseline blood tests (including tumour marker assays as appropriate) and cross-sectional imaging for diagnosis what additional assessments should be undertaken in patients with colorectal cancer and synchronous liver metastases?

NOTE: This consensus does not address preoperative liver functional assessment prior to hepatectomy as this is discussed in detail in the Surg-I-nnsbruck consensus meeting.

1. For patients of Eastern Co-operative Oncology Group staging (ECOG) 0 – 1, additional fitness tests are not required.
2. Patients should be selectively enrolled in a formal pre-habilitation programme depending on performance status.
3. Patients should selectively undergo dynamic cardiopulmonary exercise testing depending on performance status.
4. Consider the use of a validated frailty score where appropriate.
5. Nutritional status should be assessed for patients with potentially treatable metastatic colorectal cancer.

**SECTION 6: INITIAL TREATMENT PLANNING IN PATIENTS WITH POTENTIALLY RESECTABLE SYNCHRONOUS DISEASE WITH AN ELECTIVE PRESENTATION.**

Question 8: The following aspects are important in terms of assessment of the extent and distribution of the disease at presentation.

1. Presence or absence of extra-hepatic metastases should be specified.
2. In relation to thoracic metastases, number, laterality and definite or “indeterminate” should be noted.
3. In relation to liver metastases, the number of lesions should be specified.
4. In relation to liver metastases, the size of lesions should be specified.
5. In relation to liver metastases, the location within Couinaud segments should be specified.
6. Additionally, the term “juxta inflow” should be used for lesions in contact with the liver inflow.
7. Additionally, the term “juxta outflow” should be used for lesions in contact with hepatic veins.
8. Additionally, the term “juxta caval” should be used for lesions in contact with the vena cava.
9. In relation to a primary colon or rectal tumour, a radiological assessment of T and N stage should be recorded.
10. In patients with a rectal primary tumour there should be a radiological assessment of lateral lymph nodes and whether the circumferential margin is at risk.

**SECTION 7: TREATMENT – CONSIDERATIONS FOR “UPFRONT” SYNCHRONOUS SURGERY IN PATIENTS WITH POTENTIALLY RESECTABLE SYNCHRONOUS DISEASE WITH AN ELECTIVE PRESENTATION.**

**.**

Question 9: It is accepted that there is not robust evidence to guide selection of patients with synchronous disease for synchronous surgery. Patients with colorectal cancer and synchronous liver metastases may be considered for upfront synchronous resection of primary and liver metastases in some clinical settings.

1. In addition to resectable liver tumour(s) there must be a resectable primary tumour not requiring neoadjuvant systemic treatment or radiotherapy (as assessed on cross-sectional imaging).
2. If “up-front” synchronous hepatic surgery is to be considered, there must be an adequate future liver remnant (extent not specified).
3. Upfront synchronous surgery can be considered if the liver disease burden is resectable by minor hepatectomy (not otherwise specified) ± metastasectomy in addition to resection of the primary tumour.
4. Upfront synchronous surgery can be considered if the liver disease burden is resectable by major hepatectomy (hemi-hepatectomy or beyond) ± metastasectomy in addition to resection of the primary tumour.
5. Major hepatectomy should not be combined with total mesorectal excision for rectal tumour.
6. Ablative techniques (otherwise not specified) can be directed at the liver (± resection) as first-line up-front treatment.

**SECTION 7: TREATMENT – CRITERIA AGAINST UPFRONT SYNCHRONOUS SURGERY IN PATIENTS WITH SYNCHRONOUS DISEASE WITH AN ELECTIVE PRESENTATION.**

**.**

Question 10: Criteria against upfront synchronous surgery for patients with an elective presentation of colorectal cancer and synchronous liver metastases include the following:

1. Significant co-morbidity (method of assessment not specified).
2. Extrahepatic disease at presentation (M_1b_ status).
3. Peritoneal metastases at presentation (M_1c_ status).
4. (Radiological) T4 status of the primary tumour.
5. (Radiological) N1 (or beyond) status of the primary tumour.
6. Rectal tumour requiring radiotherapy, chemoradiotherapy or primary chemotherapy.
7. Rectal tumour requiring total mesorectal excision

**SECTION 7: TREATMENT – CONSIDERATIONS FOR SYSTEMIC CHEMOTHERAPY AS FIRST TREATMENT IN PATIENTS WITH AN ELECTIVE PRESENTATION OF COLORECTAL CANCER AND SYNCHRONOUS LIVER METASTASES.**

**.**

Question 11: Criteria favouring systemic chemotherapy as first treatment. This consensus refers clinicians to the current ESMO guidelines for decision making around choice of chemotherapy agent, use of combination chemotherapy, biologic agent(s) and treatment intervals. Note that this question complements question 10.

1. Performance status which precludes synchronous surgery.
2. Extrahepatic disease at presentation (M_1b_ status).
3. Peritoneal metastases at presentation (M_1c_ status).
4. Bi-lobar hepatic metastases (not otherwise specified).
5. (Radiological) T4 status of the primary tumour.
6. (Radiological) N1 (or beyond) status of the primary tumour.

**SECTION 7: TREATMENT – SYSTEMIC CHEMOTHERAPY AS FIRST TREATMENT IN PATIENTS WITH SYNCHRONOUS DISEASE AND RESECTABLE LIVER METASTASES AT PRESENTATION.**

Question 12: The type and duration of systemic treatment are discussed in detail in the ESMO and NCCN guidelines, but key aspects are discussed here. Chemotherapy backbone should be FOLFOX or FOLFIRI or FOLFOXIRI chemotherapy or (oral 5-FU equivalent) depending upon previous treatment, residual toxicity, patient preference and Performance score.

1. Standard backbone chemotherapy without monoclonal antibody should be considered.
2. Alternatively, a biologic agent (Epidermal Growth Factor Receptor Inhibitor [EGFRi]) should be added to standard chemotherapy in patients with extended RAS wild type primary tumour (left sided tumours only)
3. Or, Alternatively, a biologic agent (Epidermal Growth Factor Receptor Inhibitor [EGFRi]) should be added to standard chemotherapy in patients with extended RAS wild type primary tumour regardless of sidedness.
4. Bevacizumab may be added to the standard chemotherapy backbone
5. Only patients with a response to chemotherapy (partial response or stable disease) should proceed to liver resection.

**SECTION 7: TREATMENT – CONSIDERATIONS FOR THE BOWEL-FIRST APPROACH IN PATIENTS WITH AN ELECTIVE PRESENTATION OF COLORECTAL CANCER WITH SYNCHRONOUS LIVER METASTASES AFTER SYSTEMIC CHEMOTHERAPY.**

Question 13: Criteria favouring a bowel-first approach after systemic chemotherapy for patients with colorectal cancer and synchronous liver metastases include:

1. Symptomatic primary tumour.
2. Imminent endoscopic or radiologic obstruction
3. Resectable primary tumour with unresectable liver metastases.
4. Resection of primary tumour only in a setting where there is no availability of liver surgery.

**SECTION 7: TREATMENT – RE-STAGING AFTER INITIAL SYSTEMIC CHEMOTHERAPY IN PATIENTS WITH AN ELECTIVE PRESENTATION OF SYNCHRONOUS DISEASE.**

Question 14: Re-staging after systemic chemotherapy.

1. Re-evaluation should be considered after 8 to 12 weeks of treatment with systemic chemotherapy.
2. The liver disease should be re-evaluated by contrast-enhanced CT.
3. The liver disease should be re-evaluated by hepatobiliary contrast-enhanced liver MR where available.
4. In patients initially regarded as unresectable and treated with systemic chemotherapy ^18^FDG-PET scan should be considered before undertaking surgery in addition to CT and/or MR.
5. In patients with rectal tumours treated by chemotherapy or chemoradiotherapy, consider re-staging the primary tumour before considering hepatic resection.

**SECTION 7: TREATMENT – CONSIDERATIONS FOR THE LIVER-FIRST APPROACH AFTER SYSTEMIC CHEMOTHERAPY IN PATIENTS WITH AN ELECTIVE PRESENTATION.**

Question 15: The liver-first approach after systemic chemotherapy can be considered in the following situations:

1. When there are specific hepatic criteria such as borderline resectability which favour hepatectomy first in patients with synchronous disease.
2. This approach can be considered in patients with response after total neoadjuvant treatment including long-course chemoradiotherapy for patients with rectal tumours.
3. This approach can be used in the rare instance of patients with rectal cancer and resectable synchronous liver metastases who have a clinical complete response of the primary tumour to neoadjuvant treatment.

**SECTION 7: TREATMENT – MANAGEMENT OF “DISAPPEARING METASTASES” IN PATIENTS WITH AN ELECTIVE PRESENTATION.**

Question 16: “Disappearing metastases” are defined as liver lesions which were noted on cross-sectional imaging at baseline and are no longer noted on imaging after systemic chemotherapy.

1. The term disappearing metastases is defined in this study as lesions present on baseline contrast MR which are no longer visible on hepatobiliary contrast MR after systemic chemotherapy.
2. The presence of a “scar” on cross-sectional imaging is termed “evidence of treatment response” but if visible on hepatobiliary contrast MR, the lesion is not regarded as “disappearing”.
3. Intra-operative assessments such as ultrasound (with or without contrast) are not included in the definition of disappearing liver metastases.
4. Hepatic resection should plan to resect all sites where disease was present at baseline.
5. A “watch and wait” policy may be adopted in those patients who have a complete radiological hepatic response (disappearing metastases).
6. A bowel-first approach should be adopted in patients who have a radiological complete hepatic response to systemic chemotherapy.

**SECTION 7: TREATMENT – MANAGEMENT OF SYNCHRONOUS THORACIC METASTASES IN PATIENTS WITH AN ELECTIVE PRESENTATION OF SYNCHRONOUS DISEASE.**

Question 17: The management of thoracic metastases in a patient presenting with colorectal cancer and synchronous liver metastases:

1. The presence of definite pulmonary metastases on cross sectional imaging is M_1b_ disease and is an indication for systemic treatment as first line rather than surgery.
2. In addition to the oncologic status of M_1b_ disease, the physiologic injury consequent upon pulmonary, hepatic and bowel surgery under a single anaesthetic precludes synchronous lung, liver and bowel resection.
3. Alternatively, in specific settings, synchronous thoracic, liver and bowel surgery can be considered (if this question is answered affirmatively, please provide additional information in the comments section).
4. The opinion of a thoracic MDT about the potential locoregional treatment of pulmonary metastases should be sought before embarking on liver or bowel surgery.

**SECTION 7: TREATMENT – MINIMALLY INVASIVE SURGERY IN PATIENTS WITH AN ELECTIVE PRESENTATION OF SYNCHRONOUS DISEASE.**

Question 18: Minimally invasive surgery.

1. Minimally invasive approaches for both primary tumour and liver metastases are regarded as equivalent to the open approach.
2. Currently, there is no evidence to favour the minimally invasive approach over open surgery in the synchronous setting.
3. If a minimally invasive approach to liver resection is to be adopted, a liver resection complexity score should be used to guide selection of approach.
